# The Neurocognitive Performance of Visuospatial Attention in Children with Obesity

**DOI:** 10.3389/fpsyg.2016.01033

**Published:** 2016-07-06

**Authors:** Chia-Liang Tsai, Fu-Chen Chen, Chien-Yu Pan, Yu-Ting Tseng

**Affiliations:** ^1^Lab of Cognitive Neurophysiology, Institute of Physical Education, Health and Leisure Studies, National Cheng Kung UniversityTainan, Taiwan; ^2^Department of Recreational Sport and Health Promotion, National Pingtung University of Science and TechnologyTainan, Taiwan; ^3^Department of Physical Education, National Kaohsiung Normal UniversityKaohsiung, Taiwan; ^4^School of Kinesiology, University of Minnesota, MinneapolisMN, USA

**Keywords:** obesity, attention, visuospatial ability, event-related potential, cognition

## Abstract

The present study investigates the behavioral performance and event-related potentials (ERPs) in children with obesity and healthy weight children when performing a visuospatial attention task. Twenty-six children with obesity (obese group) and 26 healthy weight children (control group) were recruited. Their behavioral performance during a variant of the Posner paradigm was measured, and brain ERPs were recorded concurrently. The behavioral data revealed that the obese group responded more slowly, especially in the invalid condition, and exhibited a deficit in attentional inhibition capacity as compared to the control group. In terms of cognitive electrophysiological performance, although the obese group did not show significant differences on P3 latency elicited by the target stimuli when compared to the control group, they exhibited smaller P3 amplitudes when performing the visuospatial attention task. These results broaden previous findings, and indicate that childhood obesity is associated with a reduced ability to modulate the executive function network which supports visuospatial attention.

## Introduction

The prevalence of childhood overweightness and obesity has risen dramatically worldwide, and reached epidemic proportions over the past few decades (Wang and Lobstein, 2006; Liang et al., 2014). As a chronic condition, childhood obesity could be a contributor to the global burden of chronic disease, since children who are overweight or obese face a greater risk of developing various health problems, such as hypertension, cardiovascular disease, and type 2 diabetes mellitus (Reilly et al., 2003; Hannon et al., 2005). Children who are overweight are also prone to remain overweight as adults (Whitaker et al., 1997). Childhood obesity has thus become a major topic in discussion with regard to public health worldwide, due to the rise in premature obesity-related morbidities and related healthcare costs in recent decades. Moreover, some recent studies suggest that childhood obesity could not only be associated with adverse health sequelae, but also with cognitive problems (Davis and Cooper, 2011; Liang et al., 2014).

Executive functions, also called cognitive control, are a family of top–down mental processes, and this includes cognitive flexibility (i.e., response mapping, attention, or the ability to switch perspectives spatially), inhibition (e.g., response inhibitory and interference control), and working memory (Diamond, 2013). These aspects of cognition denote an ability to sustain or flexibly redirect attention, as well as inhibit inappropriate behavioral responses (Robbins, 1998). Executive functions are thus important in individuals with obesity, as they can help them to orchestrate and maintain healthy goal-directed behaviors related to food intake and physical activity (Riggs et al., 2010). Moreover, numerous experimental studies demonstrate that problems with executive functions exist in children with obesity (Cserjesi et al., 2007; Li et al., 2008; Fergenbaum et al., 2009; Lokken et al., 2009; Kamijo et al., 2012a,b, 2014; Skoranski et al., 2013; Wirt et al., 2014).

Childhood obesity has negative associations with various aspects of neurocognitive functioning, such as attention and visuospatial performance (Liang et al., 2014). Individuals with obesity show diminished functional connectivity of the middle frontal gyrus and the lateral occipital cortex with the entire brain network, the regions involved in several brain circuits signaling perceptual processes, attention, executive, and motor functions (García-García et al., 2015). Compared to healthy weight children, those with obesity show reduced attentional focus (Cserjesi et al., 2007), and even during adulthood individuals with obesity still exhibit problems with regard to attention and cognitive flexibility (Cserjési et al., 2009). In addition, more obese children/adolescents show lapses of attention (Pauli-Pott et al., 2010), and attention shifting/focus performance is associated with children’s body weight (Wirt et al., 2015). In terms of visuospatial ability, previous studies demonstrated that children with higher body mass index (BMI) (i.e., over BMI 24) exhibited greater problems in visuospatial organization (Li et al., 2008) and mental rotation tasks (Jansen et al., 2011) compared to healthy weight children. Similarly, Martin et al. (2016) also found that boys with obesity showed significantly lower performance in visuospatial skills compared with those with a healthy weight, and obesity at age 3 years continued to predict decreased visuospatial skill at age 5 years (Martin et al., 2016).

Inhibitory control includes self-control, which is related to resisting temptation and not acting impulsively, as well as interference control, which involves cognitive inhibition (i.e., abilities related to suppressing prepotent mental representations) and selective attention/attentional inhibition (i.e., inhibitory control of attention which is related to the level of perception) (Diamond, 2013). There have been a few neuroimaging studies which find that individuals with obesity have significantly less gray matter volume in the prefrontal cortex, a brain region important in response inhibition (Raji et al., 2010; Horstmann et al., 2011; Maayan et al., 2011). Many studies have thus examined the relationship between childhood obesity and inhibitory control using the Go/No Go test, and found that pediatric obesity is linked to poorer inhibitory control abilities, and the degree of inhibitory control can help to predict bodyweight in children (Pauli-Pott et al., 2010; Kamijo et al., 2012a,b; Wirt et al., 2014). These previous studies demonstrated that children with obesity showed deficits in cognitive inhibition. However, although reduced attentional ability (Cserjesi et al., 2007; Davis and Cooper, 2011; Maayan et al., 2011) and poorer attention shifting performance (Pauli-Pott et al., 2010; Wirt et al., 2015) are associated with childhood obesity, no research has yet been conducted on the inhibitory control of attention, which involves one of the interference control abilities at the level of perception (Diamond, 2013).

In the present study, a computerized serial reaction time (SRT) task (Robertson, 2007), the Posner paradigm, was used to assess the attention-related neurocognitive performances in the children with obesity. The task involves presenting a short sequence in each trial comprising a location cue followed by a target. In some trials the cue is valid, and in some trials it is not valid. The Posner paradigm requires a covert orienting of a visuospatial attention task, and is regarded as a valid and reliable measure for evaluating an individual’s capacity for different attentional control modes (e.g., alerting, orienting, and shifting) (Posner et al., 2007). Given that the Posner paradigm task allows for the manipulation of executive function demands not only on attention and visuospatial processing abilities, but also on inhibitory control of attention [attentional inhibition, as measured by the inhibitory response effect (subtracting the mean RT between valid and invalid trials)] based on the response requirements (Posner and DiGirolamo, 1998; Theeuwes, 2010), we used it to elucidate the association between childhood obesity and neurocognitive performance in the current study. The visuospatial attention task coupled with concomitant electrophysiological recording (e.g., event-related potential, ERP) provides images of cortical activity with a high temporal resolution (milliseconds), and the evaluation of the time evolution of the global brain response to cognitive processing (Babiloni et al., 2009). Among the various different visual response ERP components elicited by the Posner paradigm, the endogenous ERP P3 regarding upstream processes has been demonstrated to reflect attention-related brain activity, that is, the resource allocation necessary for attention (e.g., stimulus evaluation) (Perchet and Garcia-Larrea, 2005; Polich, 2007; Neuhaus et al., 2009). In addition, the P3 component is also involved in conflict-related brain activity, this is, the attentional resources that are allocated to efficiently inhibit a response (Jonkman et al., 2003). A previous study reported that children with obesity had smaller P3 amplitudes than normal-weight ones when given a cognitive task involving attentional processes (i.e., the oddball paradigm) (Babiloni et al., 2009). The ERP component was thus used in the current study to better understand the cognitive neurophysiological mechanism of visuospatial attention in children with obesity.

In summary, compared to normal-weight controls, children with obesity seem to have problems in attention/attentional shifting (Cserjesi et al., 2007; Pauli-Pott et al., 2010; Davis and Cooper, 2011; Maayan et al., 2011; Wirt et al., 2015) and visuospatial abilities (Li et al., 2008; Jansen et al., 2011; Martin et al., 2016). To the best of our knowledge, no ERP study has used a paradigm that directly measures the shifting of visuospatial attention in children with obesity. Additionally, although numerous studies demonstrate that children with obesity show impairments which inhibit their prepotent mental and behavioral responses, there is a lack of research with regard to the attentional inhibition of such a group. The aim of this study was therefore to investigate the relationship between childhood obesity and executive functions, involving both visuospatial attention shifting and inhibitory control of attention, using the Posner paradigm task. Based on the findings reviewed above, we hypothesized that children with obesity relative to healthy-weight cohorts would exhibit worse behavioral (i.e., slower reaction times and lower accuracy rates) performances and ERP abnormalities (i.e., prolonged P3 latencies and smaller P3 amplitudes) when performing the visuospatial attention task.

## Materials and Methods

### Participants

Fifty-two children aged 9–10 years were recruited from mainstream classrooms in urban areas of Taiwan and categorized into obese (*n* = 26; eight girls; age: 114.58 ± 3.69 months) and healthy weight (control) (*n* = 26; eight girls; age: 113.73 ± 3.85 months) groups. Children in the obese group (BMI = 27.39 ± 1.62 kg/m^2^) had a body mass index (BMI, calculated as weight/height^2^) greater than the 95th percentile for their height and weight, and children in the control group (BMI = 18.45 ± 2.23 kg/m^2^) had BMIs between the 5th and 85th percentiles according to Taiwanese norms, as seen in the related BMI-for-age growth chart (Ministry of Education, 2015). To exclude confounding effects of cardiorespiratory fitness on cognition (Hillman et al., 2008), all children had their cardiorespiratory fitness assessed using the PACER test, a multistage progressive 20-m shuttle run test, in the Brockport Physical Fitness Test Kit (Human Kinetics, Champaign, IL, USA). The number of shuttle runs was not significantly different [*t*(50) = 0.35, *p* = 0.729] between the obese (40.38 ± 25.72) and control (42.54 ± 18.32) groups. All the participants had normal or corrected-to-normal vision and were right-handed, as assessed by the Edinburgh Handedness Inventory (Oldfield, 1971). None of the children who took part in the current work had any clear signs of neurological disorders or behavioral problems, or special needs in education that would exclude them from this study. All the children were assessed using the Wechsler Intelligence Scale for Children-Revised (WISC-R), and fell within normal intelligence quotient (IQ) scores [obese: 104.56 ± 5.74, control: 106.69 ± 10.53, *t*(50) = -0.41, *p* = 0.682]. In addition, since children suffering from attention deficit hyperactivity disorder (ADHD) have shown abnormalities in behavioral response and ERPs during completion of the Posner paradigm (Perchet et al., 2001), parents and teachers were asked to complete a brief behavior rating scale (Dupaul et al., 1998) based on the DSM-IV criteria for ADHD, considering the children’s behavior patterns in the last 6 months, and verify that they did not have DSM-IV ADHD (less than six inattention and six hyperactive/impulsive symptoms). Parental education level [obese vs. control groups: 14.96 ± 2.78 vs. 15.92 ± 3.24 years, *t*(50) = 1.15, *p* = 0.256] was not significantly different between the two groups. Prior to the beginning of the experiment, each child and his/her legal guardians provided written informed assent and consent, and this study was approved by the Institutional Ethics Committee.

### The Posner Paradigm

The Posner paradigm was carried out with reference to a previous study, in which the cognitive task was used with subjects of a similar age (Tsai et al., 2009). The cognitive task was presented on a computer screen, with a fixation cross (0.5° × 0.5°) drawn in white on a black background at the beginning of the process. This served as a central fixation point, and was positioned midway between two empty white boxes (each 2 cm × 2 cm) on the same horizontal plane. The two empty boxes that were the potential locations for the target were arranged horizontally 1 cm from the fixation cross. The overall stimulus display remained on-screen until the end of the trial, except for the white fixation cross, as this was replaced by a yellow cue arrow during each trial. A trial started with a 3-s countdown followed 1000 ms later by the appearance of the two white stimulus boxes and the white fixation cross, and this was then followed 1000 ms later by the replacement of the fixation cross by the yellow cue arrow (1.5 cm in length), pointing to the right or left. After a further interval between cue onset and the appearance of the target for 350 ms, a green circle target stimulus with a diameter of 1.6 cm appeared in the center of the right or left white stimulus box. Upon detection of the green circle target the children were asked to press as quickly as possible the left “N” or right “M” button of the computer keyboard with the index or middle fingers of the dominant hand, and to avoid errors as much as possible. If the child did not respond to the target, the maximal inter-trial interval occurred 3 s after the target stimulus, and in such cases the system noted that there was a lack of response and a new trial was started. Each child completed of 180 trials, and each experimental session was divided into two consecutive runs of 90 trials each, with a 3-min break after each block, when the child was allowed to rest but remained at the workstation. Each block of 90-trials consisted of three types of trials in a random order: (i) 54 valid trials (60%), where the target appeared in the stimulus box indicated by the cue, indicating a spatially ‘valid’ condition; (ii) 27 invalid trials (30%), where the target appeared in the opposite stimulus box to that indicated by the cue, indicating a spatially ‘invalid’ condition; and (iii) nine neutral trials (10%), where the target appeared without any cue (i.e., the non-cued condition). The probability with which the targets were presented in the left or right stimulus boxes was the same. For both valid and invalid trials, the direction to which the arrow pointed, left or right, was random and equally probable. After the button was pressed, the screen cleared and the next trial started 1500 ms later. Therefore, the inter-trial interval from cue to cue was variable, depending on the speed of the child’s response.

### Procedures

Before the cognitive task test the participants and their legal guardians completed all questionnaires, as mentioned above, to ascertain if they were qualified to take part in this study, and the participants’ cardiorespiratory fitness was also assessed. On the second visit to the laboratory, the experimenter explained the procedure until the child was familiar with it. An electrocap and electro-oculographic (EOG) electrodes were attached to the child’s head and face before the test. After all the equipment had been set up, the child was asked to sit in an adjustable chair in front of a computer screen (width = 43 cm), with this linked to an IBM compatible personal computer with a stimulation system (Neuroscan Ltd., EI Paso, USA). The overall stimulus display was shown on a laptop computer screen located directly in front of the child, at face level and a distance of approximately 75 cm. All subjects simultaneously performed the Posner paradigm with concomitant electrophysiological recording. To familiarize children with the experimental procedure, a practice block of 10 trials was run before the beginning of the formal Posner paradigm test, during which they had to respond as quickly and accurately as possible, but without emphasizing one at the expense of the other (e.g., not to focus on speed to the detriment of accuracy). If they made more than 10% errors it was assumed that they could not understand the experimental procedure. In such cases the experimenter then explained the process again, and asked the child to continue practicing until they had less than 10% errors. The experimenter was seated next to the child to monitor his/her visual fixation. If the experimenter detected the child’s eye movement away from the central stimulus during the response, they gave verbal encouragement to the child to look back at the screen. The formal test was administered once the child understood the whole experimental procedure. The cognitive experiment was administered in a sound-attenuated room with dimmed lights.

### Psychophysiological Recording Methods

Electroencephalographic (EEG) activity as well as blinks and saccades were recorded from 18 sites (F7, F3, Fz, F4, F8, T3, C3, Cz, C4, T4, T5, P3, Pz, P4, T6, O1, Oz, and O2) using Ag/AgCl sintered electrodes embedded in an electrocap (Quik-Cap, Compumedics Neuroscan, Inc., El Paso, TX) according to the 10–20 system. Scalp locations were referred to linked left (A1) and right mastoids (A2), with AFz as the ground electrode. The adhesive electrodes that were placed on the supero-lateral right canthus and below and lateral to the left eye connected to the system reference for horizontal and vertical EOG (i.e., HEOG and VEOG) activity for eye movements. Electrode impedances were less than 10 kΩ. EEG data was acquired with an A/D rate of 500 Hz/channel and filter band-pass of 0.1–50 Hz, 60 Hz notch filter, and was written continuously to hard disk for off-line analysis using SCAN 4.3 analysis software (Compumedics Neuroscan, Inc., El Paso, TX, USA).

### Data Processing

The subjects’ behavioral performance was measured with the percentage of errors, as well as reaction times (RTs) to each target presentation. An error was recorded according to the following three standards: (1) anticipatory error: the participants responded sooner than 150 ms; (2) orientation error: a button-pressing error occurred (i.e., the response was not consistent with the location of the target); and (3) delay error: the participants responded more than 2000 ms after target onset. Some RTs were discarded when regarded as errors, and the rest of the RTs were grouped according to different conditions. The “strength of the inhibitory response effect” was calculated by subtracting the mean RT between valid and invalid trials (Tsai et al., 2009).

For the ERP components, the data used for the behavioral analysis was adopted to characterize the ERP elicited by the stimuli in order to truly correspond with the behavioral performance. Initially, each EEG epoch was visually inspected and discarded from those including EEG artifacts (e.g., electromyogram exceeded 100 μV peak-to-peak amplitude, VEOG, and HEOG) before averaging. The rest of the data was then averaged off-line, using a ± 100 μV automatic artifact rejection, and was constructed from both the valid and invalid conditions (excluding the non-cued condition, since this study is concerned with the strength of the inhibitory control effect, and the number of non-cued trials was small) over a 1550 ms epoch beginning 200 ms before cue stimulus onset. For target-elicited ERP components, the P3 mean amplitudes were calculated for 250–400 ms time intervals. These windows were determined from inspection of the group grand average waveforms, and were equivalent for ERP elicited by two conditions and subjects. Latencies were measured within the latency window for every child. The amplitude values for all ERP components, with reference to the 200 ms cue stimulus baseline, were determined within latency windows centered on the peak latency of the grand mean ERP.

### Statistical Analysis

For the behavioral data, since the values of the accuracy rate were not normally distributed, statistical assessment of this was carried out with the Mann-Whitney non-parametric test. The results of the separate behavioral (e.g., RTs) and cognitive electrophysiological (e.g., P3 latency and amplitude) performances were analyzed statistically by a mixed design, factorial, and repeated-measures analysis of variance (RM ANOVA), with mean RTs of accepted trials serving as the dependent variable, with Group (obese vs. control groups) as the between-subjects factor, and Condition (valid vs. invalid, excluding the non-cued condition, as this study is concerned with the strength of the inhibitory response effect) as the within-subject factor. If any interactions between the group and condition factors were found, the strength of the inhibitory response effect between the two-group comparisons was further investigated. For the ERPs data, the P3 component (e.g., latency and amplitude) was also analyzed statistically by a mixed design, factorial, and RM ANOVA, with Group (obese vs. control groups) as the between-subjects factor, and Condition (valid vs. invalid conditions) and Electrode (Fz vs. Cz vs. Pz) as the within-subjects variables. Where a significant difference occurred, Bonferroni *post hoc* analyses were performed. Estimates of effect size, partial eta-square ($\eta_{p}^{2}$), were reported for significant main effects and interactions. The significance levels of the F ratios were adjusted with the Greenhouse–Geisser correction if the assumption of sphericity was violated. The significance level was set as *p* < 0.05.

## Results

### Behavioral Performance

#### Accuracy Rate

The number of orientation errors did not show a significant difference between groups (obese: 4.19 ± 4.34, control: 5.54 ± 3.89; Mann–Whitney non-parametric test, *p* = 0.061). The numbers of anticipatory (obese: 2.54 ± 2.79, control: 3.62 ± 4.19; Mann–Whitney non-parametric test, *p* = 0.344) and delay errors (obese: 0.19 ± 0.63, control: 0.19 ± 0.57; Mann–Whitney non-parametric test, *p* = 0.987) also did not differ significantly between groups. In addition, no significant difference (obese: 0.04 ± 0.04, control: 0.05 ± 0.04; Mann–Whitney non-parametric test, *p* = 0.078) was found between groups for the error rate [i.e., (orientation error + anticipatory error + delay error)/180].

#### Reaction Time

**FIGURE 1:**
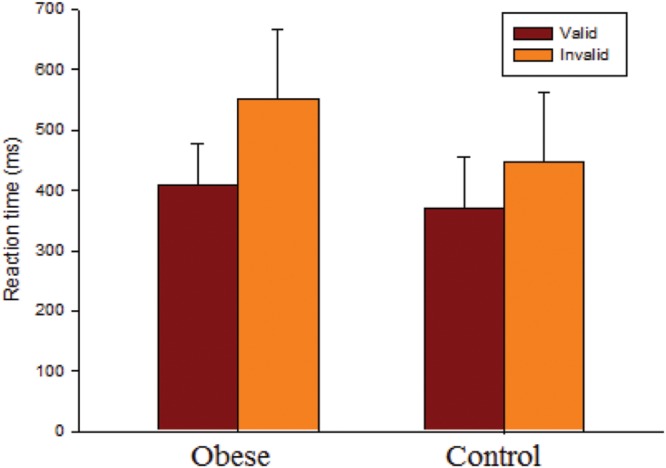
**Behavioral performance (reaction time, RT; mean ± SD) for valid and invalid conditions for the obese and control groups**.

As seen in **Figure 1**, the RM ANOVA on the RTs revealed significant main effects of Group [*F*(1,50) = 7.93, *p* = 0.007, $\eta_{p}^{2}$ = 0.14] and Condition [*F*(1,50) = 120.12, *p* < 0.001, $\eta_{p}^{2}$ = 0.71]. *Post hoc* analyses indicated that the obese group (479.58 ms) responded more slowly than the control group (408.35 ms) in both conditions, and that the valid condition (388.56 ms) showed a significant difference with regard to the invalid condition (499.37 ms) in both groups. These main effects were superseded by the Group × Condition [*F*(1,50) = 10.85, *p* = 0.002, $\eta_{p}^{2}$ = 0.18] interaction. *Post hoc* analysis showed that the obese group only responded more slowly than the control group in the invalid condition (obese: 551.63 ms vs. control: 447.11 ms, *p* = 0.002), and not the valid one (obese: 407.52 ms vs. control: 369.59 ms, *p* = 0.088). The results indicated that the average value of the strength of the inhibitory response effect (invalid RT-valid RT) (obese: 144.11 ± 93.37 ms vs. control: 77.51 ± 43.74 ms) was significantly larger in the obese group as compared to the control group.

### ERPs Performances

#### Target-P3 Latency

**FIGURE 2:**
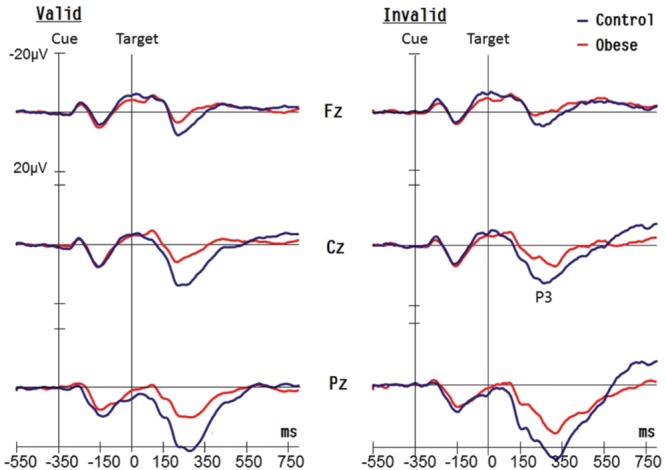
**Grand averaged waveforms (P3 components) for obese children and healthy weight (control) children for valid and invalid conditions at Fz, Cz, and Pz electrodes**.

There was no effect of Group on the latency of the target-P3 components (see **Figure 2**). An effect of Condition [*F*(1,50) = 74.06, *p* < 0.001, $\eta_{p}^{2}$ = 0.60] was observed on the latency of the target-P3, with shorter latency in valid (250.64 ms) relative to invalid conditions (295.56 ms). There was an effect of Electrode [*F*(2,100) = 47.53, *p* < 0.001, $\eta_{p}^{2}$ = 0.49] on the latency of the target-evoked P3 with the following gradient: Fz (299.39 ms) > Cz (274.49 ms) > Pz (245.41 ms). These main effects were superseded by the Condition × Electrode [*F*(2,100) = 6.68, *p* = 0.002, $\eta_{p}^{2}$ = 0.12] interaction. *Post hoc* analysis showed that the gradient Fz > Cz > Pz was found in both valid and invalid conditions.

#### Target-P3 Amplitude

As illustrated in **Figure 2**, an effect of Group [*F*(1,50) = 39.24, *p* < 0.001, $\eta_{p}^{2}$ = 0.44] was observed on the amplitude of the target-P3 response, with smaller amplitudes in the obese group (8.32 μV) in comparison with the control group (15.03 μV). There was an effect of Electrode [*F*(2,100) = 318.83, *p* < 0.001, $\eta_{p}^{2}$ = 0.86] on the amplitude of the target-P3, with the following gradient: Pz (18.93 μV) > Cz (10.97 μV) > Fz (5.12 μV). There was also a Group × Electrode [*F*(2,100) = 19.23, *p* < 0.001, $\eta_{p}^{2}$ = 0.28] interaction explained by the following gradient of amplitude, and the discrepancy was greater in the control group when compared to the obese group for the three electrodes [Pz: 27.74 vs. 14.39 μV, *p* < 0.001; Cz: 15.83 vs. 8.96 μV, *p* < 0.001; Fz: 8.16 vs. 5.89 μV, *p* = 0.049]. In addition, the interaction of Condition × Electrode [*F*(2,100) = 55.99, *p* < 0.001, $\eta_{p}^{2}$ = 0.53] also achieved a significant difference. *Post hoc* analysis showed that the gradient, Pz > Cz > Fz, was found in both valid and invalid conditions.

## Discussion

The present study examined covert orienting of visuospatial attention, either valid or invalid with target presentation, in children with obesity and healthy weight, with the aim of better understanding the visuospatial attention abilities of the obese group. Although the anticipatory, delay, and orientation errors did not differ significantly between obese and control groups, consonant with our hypothesis, the obese group relative to control group showed significantly slower RTs in the invalid condition and a significantly weaker inhibitory control of attention in the visuospatial attention task. In terms of cognitive electrophysiological performance, although the obese group, when compared to the control group, did not exhibit significant differences in the P3 latencies, they showed smaller P3 amplitudes elicited by the target stimuli. These findings show that children with obesity may not only have neuropsychological deficits in attentional inhibition, but could also have certain aberrances in visuospatial attention processing compared with controls, supporting the conclusions of previous fMRI studies that obesity is linked with dysregulated activation in a distributed network of areas involved in executive function/attention (Carnell et al., 2012; García-García et al., 2015).

Since the children with obesity showed comparable accuracy rates as the controls, the RT differences between the two groups could be attributed to the differences in processing time, and not due to any trade-off between speed and accuracy. The computerized visuospatial attention task in the current study is a SRT task which involves manual key presses and/or covert reorienting of visuospatial attention (Rosenthal et al., 2009) directed to two fixed locations in response to a target stimulus that is cued at one of the two spatial locations. The present finding suggests that children with obesity could be less efficient in dealing with the cue-target sequence supporting the previous finding that individuals with obesity showed longer RTs when performing a computerized cognitive task involving attentional responses (Babiloni et al., 2009). In addition, the obese group exhibited significantly slower RTs across all conditions relative to the control group when performing the visuospatial attention task, also supporting earlier studies investigating the relationship between childhood obesity and attentional problems (Cserjesi et al., 2007; Pauli-Pott et al., 2010; Davis and Cooper, 2011; Maayan et al., 2011; Wirt et al., 2015). For example, Cserjesi et al. (2007) found that children with obesity, compared to the control children, showed prolonged RTs on an attention endurance task, and the correlation analysis revealed a relationship between BMI/body weight and the results of the attention task in children. Nederkoorn et al. (2006) also found that more obese children/adolescents exhibited slower RTs when performing the stop-signal task needing attention requirement. Therefore, given the findings in the present and previous studies, it seems probable that the obese group showed poorer executive functions involving visuospatial attention relative to the control group.

Interference control, one of the inhibitory control abilities, includes cognitive inhibitory and attentional inhibition (Diamond, 2013). Individuals with obesity have significantly less gray matter volume in the brain areas (e.g., orbitofrontal cortex) involving response inhibition (Raji et al., 2010; Horstmann et al., 2011; Maayan et al., 2011). Previous studies have demonstrated that children with obesity showed deficits in cognitive inhibitory focus using the Go/No Go test (Pauli-Pott et al., 2010; Kamijo et al., 2012a,b; Wirt et al., 2014). The obese group in the current study also seemed to have a problem with regard to attentional inhibition, since the significantly larger RT performance in the strength of inhibitory response in this group demonstrated that these subjects’ inhibitory control of attention was worse with regard to orienting attention toward the falsely indicated location than that seen with the healthy weight children. The findings also indicate that children with obesity were much slower at modifying a movement after they had been primed to an invalid location (i.e., they were unable to complete the attentional shift as efficiently as the controls). In contrast, healthy weight children could be less reliant on the spatial information given by the cue when preparing their responses, and thus maintain attentional scanning of the whole field. Indeed, Li et al. (2008) found that children with higher BMI showed poorer performance in visuospatial organization, even controlling for parental socioeconomic status. Similar to the visuospatial attention task adopted in the present study, Pauli-Pott et al. (2010) used an incompatibility task (i.e., press left or right button depending on whether the arrow points right or left) to measure attention and the capability to resist interference and inhibit a preponderant response, and found that more obese children/adolescents showed lower inhibitory control performance due to lapses of attention. Likewise, Wirt et al. (2015) also adopted a cognitive flexibility task (i.e., press left or right button depending on the color of stimuli on the left and right sides) to assess the attention shifting/focus, and found that cognitive flexibility performance was associated with the children’s body weight. The current findings also demonstrate that children with obesity, as compared to the healthy weight controls, suffered a reduction in the time efficiency of the central processing of cognitive functions associated with the disengagement of visuospatial attention. This indicates that weight status during childhood could be related to the visuospatial attention networks that have been implicated in executive functions (Bruce et al., 2010; Carnell et al., 2012). Accordingly, based on the findings outlined above, a consensus appears to have been reached in the literature concerning the negative association between childhood obesity and visuospatial attention information processing.

P3 is an ERP component typically associated with attentional stimulus evaluations, with P3 latency being related to the speed of cognitive stimulus processing and response selection, and P3 amplitude being proportional to the amount of attentional resources allocated to a task (Polich, 2007). There was no significant difference in the P3 latencies between the two groups in the current study, suggesting that the time needed for target stimulus evaluation and detection was comparable in both groups (Perchet and Garcia-Larrea, 2000). However, the obese group exhibited smaller P3 amplitudes, despite having similar intelligence and cardiorespiratory fitness levels as those in the control group, indicating that the children with obesity exhibited less efficient allocation of attentional resources or reduced attention focus compared to the healthy weight children when performing the visuospatial attention task. However, this finding is somewhat inconsistent with Kamijo et al. (2012b), which showed no significant between-group (obese vs. healthy weight) differences in the P3 amplitudes for both the Go and No Go tasks. However, in agreement with Carnell et al. (2012) it was noted in the current study that individuals with obesity showed less activation in areas associated with object processing and attention, potentially indicating a relative absence of objective evaluation of stimuli. Similarly, Babiloni et al. (2009) used an oddball paradigm to assess the attentional cortical response, and found that the amplitude of medial prefrontal P3 sources (Brodmann area 9) was lower in the obese than normal-weight subjects in the food condition, and there was a negative correlation between the body fat percentage and P3 amplitude across conditions. These previous findings suggest that individuals with obesity show less activation in the prefrontal cortex implicated in the attention and cognitive control (Babiloni et al., 2009; Carnell et al., 2012). Nevertheless, in the present study, the effects of the between-group difference in the P3 amplitude were significant not only at the frontal cortex, but also at the central and parietal cortices when the obese and control groups performed a cognitive task involving attentional control (one of the interference control abilities). The results are partly in accordance with those in the earlier studies which found a significant discrepancy between the P3 amplitudes of obese and healthy weight children at the central (Cz) electrode when performing the auditory oddball task (i.e., children with obesity showed decreased P3 amplitude when compared to the healthy controls, Tascilar et al., 2011). In addition, when exposed to pictures of food in a eucaloric state or in response to anticipatory food, individuals with obesity showed obviously discrepant activation in the parietal lobe as compared to the lean individuals (Stice et al., 2008; Tregellas et al., 2011). The previous and present findings may thus, at least partly, reflect that individuals with obesity show impairments in the neural activity of the frontoparietal cortices with regard to activation, and diminished functional network connectivity in the brain areas involved in several brain circuits signaling perceptual processes, attention, executive and motor functions (Stice et al., 2008; Carnell et al., 2012; García-García et al., 2015).

It is worth noting with regard to attentional networks that obesity is related to a decrease in striatal dopaminergic receptors (Wang et al., 2001), and that dopaminergic transmission plays an important role in attentive neural processing (e.g., the strength of the P3 signals) (Neuhaus et al., 2009). Based on the present and previous findings, as presented in the paragraph above, the attenuated P3 amplitudes found for the obese group could be signs of weaker attentive neural processing due to fewer striatal dopaminergic receptors. In addition, the cognitive task adopted in the present study is a sequential task. Children with obesity performing the visuospatial attention task during the test had to learn a higher-order association in the SRT task which involves a distinct cortico-cerebellar/cortico-striatal network, with activation in the inferior parietal lobule identified with encoding an effector-independent description of successive locations (Grafton et al., 1998; Seidler et al., 2005). Poorer cognitive electrophysiological performance (e.g., P3 amplitude) in children with obesity in the present study seems to imply that such a group seems to have deficits in these neural networks. Also, the P3 amplitude and cognitive resource allocation may more or less reflect the physiological processes related to the RTs (Muller and Knight, 2002), and since the children with obesity in the present study exhibited smaller P3 amplitudes, this might result in longer RTs. Moreover, P3 has been suggested as an inhibition component in children (Jonkman et al., 2003), and children with higher BMI are associated with less activation in the brain regions involved in inhibitory control (Batterink et al., 2010). The smaller P3 amplitudes found in the current study could also partly explain the poorer attentional inhibition in children with obesity.

Although some potentially confounding factors (e.g., parental education level and cardiorespiratory fitness) which could mediate the obesity-cognition association were rigorously controlled in the present study (Hillman et al., 2008; Li et al., 2008), there are still some potential limitations to its cross-sectional study design. Dual-process theories of attention (Corbetta and Shulman, 2002) propose that orienting of attention is controlled by dorsal and ventral networks (Butler et al., 2009), which might represent the endogenous, goal-directed attention orienting system and the exogenous, stimulus-driven attention orienting system, respectively (Perry and Zeki, 2000; Corbetta and Shulman, 2002). In the current study, the endogenous orienting task [i.e., a high probability (i.e., 60%) of valid precues] was adopted to give rise to the facilitatory effect (Muller and Rabbitt, 1989; Rafal and Henik, 1994). Therefore, children with obesity displayed a deficit in volitional/intentional orienting of visual attention, that is, in the dorsal attention network. As yet it remains unclear whether children with obesity also display a visuospatial attention deficit in shifts of automatic (exogenous) attention. In addition, individuals with metabolic syndrome (MS) have showed an approximately fourfold increased risk of lowered cognitive performance after adjusting for insulin resistance (IR) relative to those without MS (Fergenbaum et al., 2009). Children who are overweight/obese are prone to have dyslipidemia and MS (Casavalle et al., 2014). Children with obesity and IR show significantly different electrophysiological performance compared to those without IR when doing the auditory oddball task, suggesting that IR is an important risk factor leading to cognitive dysfunction in such children (Tascilar et al., 2011). Therefore, one avenue for future research is to examine the possibility of an interaction among childhood obesity, MS, IR, and neurocognitive functions. Further, obesity is determined by BMI and is associated with an excessive accumulation of peripheral fat. Although actual body fat content could not be reflected by BMI percentiles as measured from body weight (Wirt et al., 2014), in general, BMI percentile is a clinically meaningful measure for population research (Reilly et al., 2003), which enables comparability with other studies. Despite poorer performance in visuospatial attention in children with higher BMI in the present study, BMI may sometimes underestimate the relationship between cognition and weight status during childhood. Therefore, how the role of fat mass influences the relationship between obesity and executive functions in children remains an open question. Lastly, besides genetic predisposition, some life risk factors, such as preferences for a sedentary lifestyle (e.g., low levels of physical activity) and poor dietary behaviors (e.g., overeating or over-consuming fat or sugar) are demonstrated to contribute to childhood obesity (Reilly et al., 2005; Braet et al., 2007; Iannotti and Wang, 2013) which could impair cognitive functions (Bechara, 2005; Verdejo-Garcia et al., 2006). In the current study, the obese group exhibited similar cardiorespiratory fitness to the healthy weight group, which implies comparable levels of physical activity between the two. As such, the issue as to whether deficits in inattention and attentional inhibition are the underlying mechanism of overeating in children needs to be investigated.

## Conclusion

Children with obesity showed poorer behavioral (e.g., slower RTs and larger values of the strength of the inhibitory response effect) performances and aberrant neural activity (e.g., smaller P3 amplitudes) associated with cognitive information processing when doing the visuospatial attention task in the present study. Previous studies mostly examined childhood obesity with regard to the health consequences, such as diabetes, cardiorespiratory diseases, hypertension, or future risk of adult obesity. In contrast, only few studies have explored the possible relation between childhood obesity and cognitive functions, specifically with regard to executive function tasks. Executive function is responsible for adjusting behavior in relation to a situation which requires individuals to resist temptation, as well as for transmissions between the internal world and environmental challenges (Norman and Shallice, 1980/2000). The findings of the current study extend those of previous works, and imply that deviant cognitive processing should also be taken into account as an obesity-related health issue, and thus how to treat both conditions (i.e., obesity and executive function impairment), rather than obesity in isolation, is an important issue in clinical practice.

## Author Contributions

Dr. C-LT designed the study, wrote the protocol, and the first draft of the manuscript. Dr. F-CC analyzed the data. Dr. C-YP helped explain results. Mrs. Y-TT helped collect data.

## Conflict of Interest Statement

The authors declare that the research was conducted in the absence of any commercial or financial relationships that could be construed as a potential conflict of interest.
